# Neural progenitors, patterning and ecology in neocortical origins

**DOI:** 10.3389/fnana.2013.00038

**Published:** 2013-11-12

**Authors:** Francisco Aboitiz, Francisco Zamorano

**Affiliations:** ^1^ Departamento de Psiquiatría, Facultad de Medicina y Centro Interdisciplinario de Neurociencia, Pontificia Universidad Católica de ChileSantiago, Chile; ^2^ División Neurociencia de la Conducta, Centro de Investigación en Complejidad Social, Facultad de Gobierno, Universidad del DesarrolloSantiago, Chile

**Keywords:** antihem, cortical hem, dorsal ventricular ridge, intermediate progenitors, nidopallium, Pax6, subventricular zone, reelin

## Abstract

The anatomical organization of the mammalian neocortex stands out among vertebrates for its laminar and columnar arrangement, featuring vertically oriented, excitatory pyramidal neurons. The evolutionary origin of this structure is discussed here in relation to the brain organization of other amniotes, i.e., the sauropsids (reptiles and birds). Specifically, we address the developmental modifications that had to take place to generate the neocortex, and to what extent these modifications were shared by other amniote lineages or can be considered unique to mammals. In this article, we propose a hypothesis that combines the control of proliferation in neural progenitor pools with the specification of regional morphogenetic gradients, yielding different anatomical results by virtue of the differential modulation of these processes in each lineage. Thus, there is a highly conserved genetic and developmental battery that becomes modulated in different directions according to specific selective pressures. In the case of early mammals, ecological conditions like nocturnal habits and reproductive strategies are considered to have played a key role in the selection of the particular brain patterning mechanisms that led to the origin of the neocortex.

## INTRODUCTION

Mammals display an accumulation of evolutionary acquisitions that include viviparity, lactation, major cranial and postcranial skeletal reorganizations, respiratory and metabolic innovations, changes in sensory capacities, and particularly the acquisition of a large brain, featuring a unique six-layered neocortex. Like other innovations, once acquired, the neocortex remained notably conservative, showing a highly maintained columnar and laminar architecture in all mammals studied (Nieuwenhuys, 1994; Rakic, 2009). Some incipient features of cortical organization can be observed in limbic structures like the hippocampus or the olfactory cortex of mammals, that resemble more adequately the cortical organization observed in present day reptiles. Nonetheless, the neocortex is one of those characters in which no obvious intermediate structures can be seen between its six-layered radial organization and the three-layered, tangentially organized reptilian cortex (or the mammalian olfactory cortex and hippocampus; Aboitiz and Montiel, 2007a). Partly for this reason, the establishment of homologies between the mammalian and the nonmammalian brains has been a matter of long and intense scientific debate for about a century.

What developmental events were involved in the generation of the neocortex is a fundamental question of evolutionary neuroscience. At first instance, it may seem to have required major transformations in the neural progenitor pools, cellular migration and establishment of connectivity. However, more detailed observations have revealed a fundamental similarity in the embryological processes underlying brain development in all vertebrates studied (Striedter, 1997; Puelles et al., 2000; Puelles, 2001, 2011: Brox et al., 2004; Aboitiz and Montiel, 2007a,b, 2012; Medina and Abellán, 2009; Aboitiz, 2011). Here we propose that the expansion and differentiation of the mammalian brain relies on highly conserved neurodevelopmental mechanisms, that have been differentially modulated in distinct lineages through natural selection for specific perceptual and behavioral capacities. Thus, a basic parallelism can be found in the brain expansion of mammals and that of the sister amniote lineage, the sauropsids (which includes reptiles and birds). Nonetheless, there are proposed to be differences in the regulatory systems that participate in brain patterning, associated to diverging behavioral and ecological adaptations (Aboitiz, 2011; Aboitiz and Montiel, 2012).

We will review some aspects of cortical development in mammals, and will make a comparison with what is known about the brains of reptiles and birds, including recent evidence that increasingly points to a common understanding of the evolutionary and developmental processes involved in the origin of the mammalian and the sauropsidian brains. First, the principal features of the mammalian neocortex will be discussed, followed by a comparison with the brains of other amniotes, highlighting the controversies on presumed brain homologies between mammals and sauropsids. Then, we will offer a proposal that in our view accounts for many of the apparent discrepancies that have been raised in this subject. Basically, the point is that brain growth relies on the expansion of different embryonic components in mammals and sauropsids (dorsal pallium vs. ventral pallium, respectively), but these non-homologous regions have recruited conserved genetic and developmental mechanisms for progenitor amplification and neural differentiation (Aboitiz, 2011). Thus, although the expanding brain regions are not strictly homologues in birds and mammals, there is a basic conservation of the developmental pathways that are activated in each of them. Finally, we will present a unifying perspective in which coordinated anatomical, neurobiological and behavioral factors conflated in the selection of modulatory mechanisms that provided a differential regulation of patterning centers, neurogenesis and neural differentiation in the origin of the neocortex. This perspective fits current Darwinian interpretations of the role of ecology and behavior in the selection of distinct developmental pathways (Lister, 2013; Spence et al., 2013).

## THE NEOCORTEX: DISTINCTIVE FEATURES

The anatomical organization of the mammalian neocortex is unique among vertebrates. Although basic mechanisms controlling the proliferation of progenitors, the radial migration of excitatory neurons and the tangential migration of most inhibitory neurons from the medial ganglionic eminence may be shared with other vertebrates (Aboitiz, 2011), the neocortex differs conspicuously from comparable structures of other vertebrates in the radial organization of clonally related columns of excitatory neurons, that arise in development via migration along a radial glia that is also a progenitor cell (Noctor et al., 2001; Torii et al., 2009; Yu et al., 2009; Li et al., 2012; Malatesta and Götz, 2013). Along the developing column, cells differentiate in six horizontal layers that provide the neocortex with its laminated appearance. Furthermore, the neurogenetic sequence is inside-out, that is, it differs from many other structures in that early produced neurons make up the deepest layers, and late-produced neurons have to migrate past layers of early born neurons to end up in successively more superficial layers (for reviews, see Rakic, 2009; Aboitiz and Montiel, 2012).

Another peculiar aspect is that during embryogenesis, a transient structure develops before the adult neocortex, called the preplate, consisting of tangentially migrating Cajal-Retzius cells that secrete the glycoprotein reelin on the most superficial cortical layer (Aboitiz et al., 2005; Molnár et al., 2006). Cajal-Retzius neurons develop a dense axonal plexus in the superficial preplate, which serves to define the limits of the marginal zone or future layer I of the neocortex. These cells usually express the factor p73 and originate mostly in the dorsomedial aspect of the cerebral hemisphere (the cortical hem), but also in the lateral hemisphere (the antihem), in frontal regions of the hemisphere and in the ventral thalamus, and populate the developing neocortex from different directions (Meyer, 2010; Puelles, 2011). Interestingly, the positioning of Cajal-Retzius cells in the embryonic cortex is regulated by the endfeet of radial glia (Kwon et al., 2011) and by meningeal derived factors (Borrell and Marín, 2006). Conversely, reelin activates Notch function in radial glia, accelerating neurogenesis (Lakomá et al., 2011).

Cajal-Retzius neurons are important for the establishment of the inside-out neurogenetic gradient in the neocortex, as the reeler mouse displays an abnormal outside-in neurogenetic sequence (although the defect is not homogeneous along the cerebral cortex and also affects limbic regions; Boyle et al., 2011). Current interpretations of reelin function imply that it promotes adhesion of migrating neurons to the extracellular matrix by activation of integrin alpha 5 beta 1 (Sekine et al., 2012). Furthermore, reelin signaling, mediated by Dab1, becomes activated in the very last stages of neuronal migration, during radial glia-independent translocation (Franco et al., 2011). Reelin-producing Cajal-Retzius neurons are present in the brains of other nonmammals like reptiles and birds, but in mammals the production of these cells underwent a massive amplification (Bar et al., 2000; Nomura et al., 2008, 2009). Nonetheless, artificial amplification of reelin expression in the developing chick brain induces a strong radial orientation of radial glia and a highly polarized vertical shape of migrating neurons (Nomura et al., 2008).

In the preplate, below the Cajal-Retzius layer, there is a deeper layer consisting of diverse cell types that arise by both radial and tangential migration. With the arrival of the neurons that will make the adult neocortex, the preplate becomes split into a superficial marginal zone (future layer I), containing the Cajal-Retzius cells, and a subplate that contains the elements that were located in the deep preplate. The future neocortical neurons arrange between these two layers, in the so-called cortical plate. Subplate neurons make up a transient layer that serves to guide and maintain thalamocortical axons while the definite cortical plate (adult neocortex) maturates (Molnár et al., 2012). Subplate axons establish complex but short living circuits with thalamocortical axons and with neurons of the developing cortical plate, until the latter become able to receive these axons. Removal of the subplate can have profound effects on the circuitry of the neocortex, indicating that it is a crucial regulator of neocortical plasticity (Kanold and Luhmann, 2010). The subplate is barely discernible in marsupials and increases in complexity as the neocortex expands in size in different species (Aboitiz et al., 2005; Montiel et al., 2011). In monotremes, a subplate-like structure has been identified in the lateral hemisphere that is traversed by thalamocortical axons (Ashwell and Hardman, 2012). In reptiles, subplate markers appear scattered in the superficial cell layer of the turtle dorsal cortex, while markers of the deep neocortical layer VI are found in the deep cellular layer of the dorsal cortex. GABAergic preplate-like neurons are also found in the deep internal plexiform layer of the turtle dorsal cortex (Montiel et al., 2011). A current evolutionary interpretation of the mammalian subplate is that it comprises both ancestral components that are scattered in the developing reptilian cortex, and newly acquired ones as the neocortex has become increasingly complex (Aboitiz et al., 2005; Montiel et al., 2011).

**FIGURE 1:**
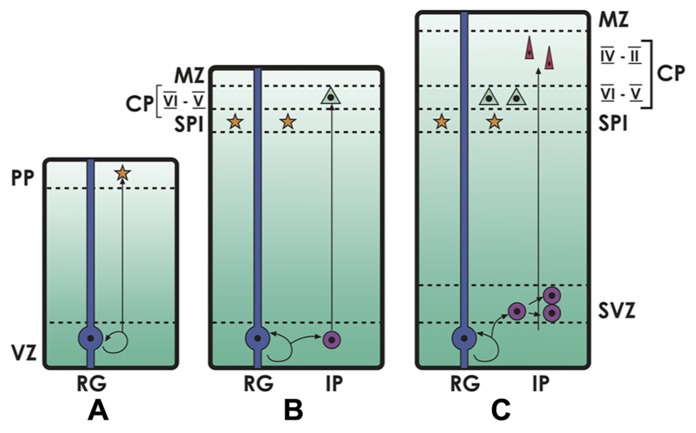
**Neocortical development.** The deep ventricular zone (VZ) and the subventricular zone (SVZ) are the compartments where cell proliferation takes place. **(A)** In early cortical development, primary neural progenitors or radial glia (RG) in the VZ divide and give rise to early neurons that migrate to the preplate (PP), and then make up the embryonic subplate (SPl). **(B, C)** Later in development, radial glia generate intermediate progenitors (IP), that keep dividing and producing neurons into the emerging cortical plate (CP, future layers VI–II of the neocortex), in an inside-out gradient where deep layers (VI–V) are formed first and mostly derive from progenitors in the VZ, and superficial layers (IV–II) are formed later, deriving from progenitors in the SVZ. The more superficial layer (Layer I) is the remnant of the embryonic marginal zone (MZ), in which Reelin-producing Cajal-Retzius neurons are located.

The cortical plate that grows between the marginal zone and the subplate, making up the future cortical layers II–VI, develops in the already described inside-out sequence, with deep layer VI forming first, then layer V above it, then layer IV and finally the superficial layers III and II (layer I, the marginal zone, remains largely free of neurons after Cajal-Retzius cells disappear in late development, together with the subplate; for reviews, see Aboitiz et al., 2003; Rakic, 2009). Radial glia, once beleived to represent only a scaffolding for neuronal migration, have been recognized in the last years as the main neural stem and progenitor cells in development, differentiating into many cortical cell types, including excitatory neurons, astrocytes and oligodendrocytes (Malatesta and Götz, 2013). On the other hand, most inhibitory neurons originate in the subpallium and populate the developing cortex via tangential migration (Anderson et al., 1997). Radial glia has shown to be highly diverse, some producing both glia and neurons, others only glia and others only neurons (Malatesta and Götz, 2013). A recent study identified a population of radial glia that is committed to produce only neurons to the superficial layers IV-III-II (Franco et al., 2012). In the deepest ventricular zone (VZ) of the hemisphere, early progenitors divide symmetrically, increasing their number, but also make up asymmetrical divisions that give rise to a self-renewing progenitor and to a cell that differentiates into a neuron, making up the earliest radial components of the subplate and the cortical plate (deep layers). Later in development, these asymmetric divisions generate one intermediate progenitor that may remain in the VZ and migrate contributing to deep neocortical layers; or may remain above the VZ, in the subventricular zone (SVZ), and keeps dividing for one or more cell cycles, producing mostly late-born, superficial cortical neurons (see **Figure 1**; Tarabykin et al., 2001; Farkas and Huttner, 2008; Pontious et al., 2008). The growth of the SVZ has been associated with neocortical expansion both in development and across species (Farkas and Huttner, 2008; Pontious et al., 2008), and appears to underly the developing neocortex of all mammals, including marsupials and monotremes. On the other hand, a SVZ containing intermediate progenitors is still lacking or is minimal in most reptiles, but appears in the subpallium of crocodiles (phylogenetically close to birds) and is maximally expressed in the embryonic avian nidopallium. The SVZ is also present in the hyperpallium of some birds who have developed this structure, possibly associated to binocularity (see below; Charvet et al., 2009; Cheung et al., 2010; Heesy and Hall, 2010; Aboitiz, 2011; Ashwell and Hardman, 2012).

## COMPARISON WITH SAUROPSIDIAN BRAINS

**FIGURE 2:**
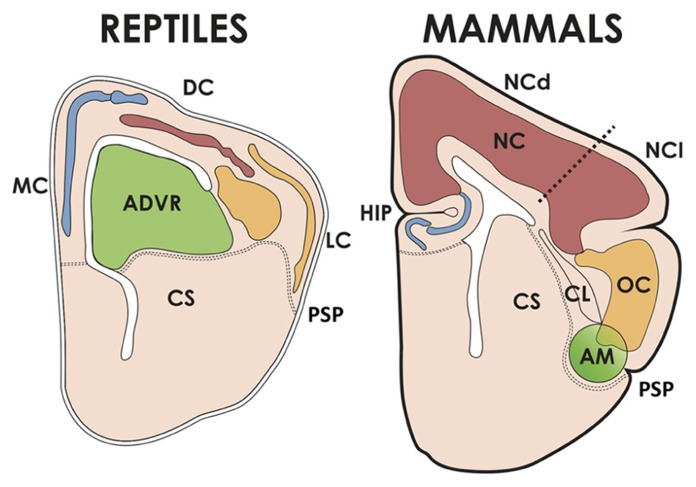
**The cerebral hemispheres of reptiles and mammals.** The pallium of reptiles has medial/dorsomedial (MC), dorsal (DC, corresponding to the avian hyperpallium) and lateral (LC) cortices; and a dorsal ventricular ridge, whose anterior part (ADVR) corresponds to the avian nido and mesopallium. The MC of reptiles corresponds to the hippocampus (HIP) of mammals, and the LC is homologous to the mammalian olfactory cortex (OC). The mammalian neocortex (NC) comprises two moieties, one dorsal (NCd, receiving lemnothalamic somatosensory and visual inputs), and one lateral (NCl, receiving auditory and visual collothalamic inputs). AM, pallial amygdalar formation, CL, claustrum, CS, corpus striatum, PSP, pallial-subpallial boundary.

The architecture of the mammalian neocortex differs significantly from brain structures that are observed in other amniotes. We will first make a brief account of the organization of the reptilian and avian brains in order to provide sufficient background for the discussion. The neocortex and other structures to be commented here belong to the pallium, i.e., the “roof” of the cerebral hemisphere or telencephalon, which is separated from the more ventral subpallium by the pallio-subpallial boundary in the lateral telencephalon (see **Figure 2**). In mammals, the pallium is further subdivided into the medial pallium (hippocampus), dorsal pallium (neocortex), lateral pallium (dorsal olfactory cortex and other structures like the dorsolateral claustrum and parts of the insular region), and a newly described subdivision between the lateral pallium and the subpallium, termed the ventral pallium (in mammals, this gives rise to the pallial claustroamygdaloid complex, olfactory bulbs and ventral olfactory cortex). In all species, the ventral pallium is characterized by lack of expression of Emx1 (a marker of all other pallial subdivisions), and the strong expression of Lhx9 in the ventricular surface. The gene Pax6 is expressed in the VZ of all pallial regions and a small region of the subpallium (for further details, see Fernández et al., 1998; Puelles et al., 1999, 2000; Medina and Abellán, 2009; Medina et al., 2011). While the correspondences of the medial pallium with the hippocampus, and of the lateral pallium with the olfactory cortex are conserved in all amniotes, there has been controversy about the homologies of the dorsal and ventral pallial regions across species (Northcutt, 1969; Northcutt and Kaas, 1995).

In reptiles, pallial cortical structures are located in the medial pallium (medial and dorsomedial cortex, comparable to the hippocampal formation), dorsal pallium (dorsal cortex) and lateral pallium (lateral or olfactory cortex; **Figure 2**). These cortical regions display a 3-layer organization as opposed to the 6 layers of the neocortex, which makes the reptilan cortex more similar in structure to the mammalian hippocampus and olfactory cortex (Aboitiz et al., 2003). Furthermore, the reptilian cortex is quite small as compared to the expansive mammalian neocortex. Nonetheless, reptiles possess the dorsal ventricular ridge (DVR), an intriguing nuclear component that bulges into the cerebral ventricle and undergoes a significant but limited expansion, yielding an about two-fold increase in hemisphere size with respect to amphibians (Northcutt, 2013). The DVR is subdivided into an anterior component (ADVR) receiving thalamic sensory input (mainly visual and auditory), and a posterior component (PDVR) that projects to hypothalamic nuclei and has been compared to parts of the amygdalar system of mammals. In birds, there is a well differentiated hippocampus, and the equivalent to the reptilian dorsal cortex has been claimed to be the hyperpallium (mentioned in the section above); finally the lateral or olfactory cortex is not very well developed. The avian equivalent to the ADVR corresponds to the nidopallium (also mentioned above; this includes a visual receiving nucleus called entopallium and an auditory region called field L), and the mesopallium. The nidopallium and the ventral/posterior DVR (arcopallium in birds), as well as parts of the olfactory cortex and olfactory bulb, have been found to derive from the embryonic ventral pallium referred to above, while the mesopallium has a lateral pallial origin (Puelles et al., 1999, 2000; Medina and Abellán, 2009; Medina et al., 2011).

The comparison of the avian nidopallium/mesopallium with mammalian structures has been a matter of controversy for about a century. While some early anatomists and embryologists associated the ADVR/nidopallium to the mammalian amygdalar complex (Holmgren, 1922; Källén, 1951), calling both structures the hypopallium, others considered that it belonged to the corpus striatum in the subpallium (Ariëns et al., 1936). In the 1960s, hodological and neurochemical evidences strongly pointed to a pallial origin of this structure (Karten, 1968, 1969; Parent and Olivier, 1970). Furthermore, Karten (1968; 1969) identified two kinds of sensory projections to the pallium of amniotes. In the first kind -now called lemnothalamic-, sensory projections ascend directly via lemniscal pathways to thalamic nuclei that relay this input to the pallium. In the second class of ascending projections -called collothalamic- there is a relay in the mesencephalon before reaching the thalamus and then the pallium (Butler, 1994a, b). According to this definition, the somatosensory projection is lemnothalamic and the auditory projection is collothalamic. In the visual system, two pathways were described, one lemnothalamic (receiving ascending retinofugal projections) and the other collothalamic (receiving ascending tectofugal projections). In Karten’s and colleagues account, both the DVR/nidopallium of sauropsids and the auditory and visual extrastriate neocortex of mammals receive collothalamic projections, while the sauropsidian dorsal cortex/hyperpallium and the mammalian somatosensory and primary visual neocortex are associated to the lemnothalamic pathways. Based on these similarities, Karten and collegues proposed that the core sensory processing circuits of the avian nidopallium were homologous to those found in the laminated auditory and extrastriate visual neocortex in mammals. On the other hand, the equivalent circuits in the hyperpallium of birds were proposed to be homologous to those in the somatosensory and primary visual cortices (Karten, 1968, 1969, 1991, 1997, 2013; Reiner, 2009). For example, in the case of the extrastriate visual cortex of mammals, a circuit involving thalamic recipient layer IV, then layers II-III and finally the output deep cortical layers V-VI was homologous to a circuit encompassing the entopallium (a subdivision od the nidopallium), the general nidopallium and the arcopallium in birds (the avian homologue to layer IV of auditory cortex is proposed to correspond to a subsector of the region termed field L in the nidopallium; Karten, 1969, 1991).

Other authors have argued that the processing circuits of the DVR/nido-mesopallium arise from a different embryonic territory (ventral or lateral pallium) than those of the neocortex, which originates from the dorsal pallium, like the reptilian dorsal cortex and the avian hyperpallium. This has strongly challenged the homologies proposed by Karten and collegues (Aboitiz, 1992, 1995; Striedter, 1997, 2005; Fernández et al., 1998; Puelles et al., 1999, 2000; Striedter and Keefer, 2000; Puelles, 2001, 2011; Medina et al., 2004, 2011; Medina and Abellán, 2009 for a recent review, see Alfano and Studer, 2013) Furthermore, separate and distinct medial, dorsal, lateral and ventral pallial subdivisions have been also described in amphibians and fish (Puelles, 2001, 2011; Brox et al., 2004; Northcutt and González, 2011). It follows that the common amniote ancestor had a likewise parcellated pallium, which prescribes non-homology between the (dorsal pallial) neocortex of mammals and the (ventral pallial) nidopallium of birds. In order to provide ventral pallial cells to the neocortical circuits described above (as Karten’s hypothesis implies), a massive neuronal migration from the ventral pallium to the dorsal pallium would be required, something that has not been observed. Nonetheless, in mammals, embryonic ventral pallial excitatory neurons derived from progenitors expressing Dbx1, reach the developing neocortex to disappear shortly after birth (Teissier et al., 2010; discussed in Puelles, 2011). Of note, these transient neurons contribute to maintaining the proliferative state of cortical precursors and to the development of superficial neocortical layers (Teissier et al., 2012).

Considering the lack of evidence for tangential migration, some supporters of Karten’s views proposed that in early amniotes, the collothalamic-recipient territory was either undifferentiated or of ventral pallial origin, and became transformed into a dorsal pallial region in mammals (while in sauropsids it differentiated into, or remained as a ventral pallial territory; Butler, 1994a,b; Reiner, 2000, 2009, 2013; Molnár and Butler, 2002a, b). However, there is no comparative evidence of a transformation process, and no undifferentiated territory has been identified in amphibians or in reptiles that might have been transformed into ventral or dorsal pallium (Brox et al., 2004). Furthermore, this alternative requires the specification of a mechanism by which collothalamic afferents remained in this site despite a major transformation in the adult phenotype.

A crucial issue in Karten’s and followers hypothesis is their proposal that the auditory- visual extrastriate neocortex and the nidopallium receive homologous collothalamic sensory afferents. On the other hand, Bruce and Neary (1995) and later Puelles et al. (2005) and Puelles (2001, 2011), argued that mammalian collothalamic projections to the neocortex are not homologous to the collothalamic projections to the nidopallium of birds, as they arise from non-homologous thalamic nuclei. In this view, thalamic projections to the mammalian auditory/visual extrastriate neocortex would have arisen independently from those to the avian nidopallium. Mammalian homologues to the sauropsidian collothalamic projections are claimed to remain, but directed to the ventral pallial basolateral amygdala. However, these projections are considered by other authors to differ profoundly from those terminating in the avian nidopallium (Reiner et al., 2005; Reiner, 2013). Intense arguments have come back and forth in this issue, which to be fair has not been settled yet (see reviews in Aboitiz and Montiel, 2007a, 2012; and Aboitiz, 2011).

A third possibility is that the so-called collothalamic projections were re-routed from the ventral pallium into the emerging neocortex in early mammals (Aboitiz, 1995, 2011; Aboitiz and Montiel, 2007a; see also Puelles, 2001). In this case, there would be a subcortical homology of the sensory pathways but their final site of termination would be different in mammals and sauropsids by virtue of a displacement of the axonal pathways. In this context, we have suggested that the transient ventral pallial neurons that populate the developing neocortex (Teissier et al., 2010, 2012) might have contributed to re-direct collothalamic afferents that in ancestral amniotes reached the ventral pallium, into the expanding neocortex of early mammals (Aboitiz and Montiel, 2012; see also Alfano and Studer, 2013).

## PALLIAL NEURONAL TYPES AND PROLIFERATIVE PROCESSES IN AMNIOTES

Despite originating from different pallial components, the mammalian neocortex and the sauropsidian nidopallium/DVR have long been claimed to serve similar sensory and behavioral functions. The question then arises as to what extent there are similarities in the neural processing circuits in these two expanding brain regions in both animal groups, and whether there are common phenotypes expressed in their neuronal components, even if such regions may not be homologous. Nonetheless, it is important to compare developmentally equivalent regions across species as well, in order to distinguish the possibilities that the shared signatures correspond to ubiquitous pallial markers that are found throughout the pallium, or to the result of phenotypic convergence in two otherwise different brain components.

Several recent findings indicate that there is expression of superficial and deep neocortical markers in the corresponding dorsal cortex and in other regions of the reptilian and avian brains (see **Table 1**). For instance, Luzzati et al. (2009) observed expression of Tbr1, doublecortin and polysialylated neural cell adhesion molecule in the dentate gyrus (hippocampus) and the superficial layers of neocortical and olfactory cortical areas of mammals, and in medial, lateral, and ventral pallial regions of the lizard (medial cortex-hippocampus, lateral cortex, and DVR/nucleus sphericus respectively). According to these authors, these markers were co-opted for the origin of late-produced neuronal types in the DVR of sauropsids and in the neocortex of mammals. Additionally, the gene Brn2, active in the superficial neocortical layers II-III, is expressed in the hyperpallium apicale, mesopallium and part of the nidopallium of the quail (Nomura et al., 2008). On the other hand, the gene Er81, active in the hippocampus, the neocortical layer V, amygdala and striatum of the mouse, is expressed in the hippocampus, parahippocampal region, arcopallium and striatum of birds (Nomura et al., 2008). More recently, Suzuki et al. (2012) observed a series of upper and mid-neocortical layer markers (layers II-IV; Satb2, Cuz2, Met2c, and FoxP1) in the mesopallium of the chick, and lower layer neocortical markers (mainly layer V; FoxP1, Er81, Fezf2, Ctip2) in the parahippocampal region.

**Table 1:** ummary of the main findings of recent reports describing expression of different markers in distinct brain regions in mammals and birds.

	Medial Pallium	Dorsal pallium				Lateral pallium	Ventral pallium		Subpallium
	Hippocampus	Hyperpallium				Mesopallium	Nidopallium	Arcopallium	Striatum
		HA	IHA	HI	HD		entop.-fl
Brn2 (NCx LII-III)
(Nomura et al., 2008)
Quali
Satb2, Cux2, Mef2c, FoxP1 (NCx LII-IV)
(Suzuki et al., 2012)
Chicken
EAG-RORB (NCx LIV-LAA)
(Dugas-Ford et al., 2012)
Chicken, Zebrafinch
Nrn1, Myo16, Rorb, Fam19a2,
Stoml2 (NCx LIV)
(Belgard et al., 2013)
Quail
Er81 (HP, NCx LV, BLA, STR)
(Nomura et al., 2008)
Quail
(Dugas-Ford et al., 2012)
Chicken, Zebrafinch
Er81, Fezf2, Ctip2 (NCx LV)
(Suzuki et al., 2012)
Chicken
(Chen et al., 2013; Jarvis et al., 2013)
Zebrafinch

*Abbreviations: BLA, Basolateral amygdala; ENTOP.-FL, Entopallium and Field L; HA, Hyperpallium apicale; HD, Hyperpallium densocelulare; HI, Hyperpallium intercalatum; HP, Hippocampus; IHA, Interstitial hyperpallium apicale; LAA, Lateral anterior amygdala; NCx, Neocortex; LII-LIII, Neocortical layers II-III; LII-LIV, Neocortical layers II-IV; LIV, Neocortical layer IV; LV, Neocortical layer V; STR, Striatum.*

The above evidence has suggested to some of these authors (Nomura et al., 2008, 2013a,b; Suzuki et al., 2012) that the phenotypic differentiation sequence occurs in different axes in mammals and sauropsids. According to them, in the mammalian neocortex, phenotypic segregation takes place radially, with different types of neurons originating in the same region (and likely from the same progenitor) of the VZ, but in a specific temporal sequence (early born neurons make up deep layers, and late born neurons make up superficial layers). On the other hand, in birds, the differentiation sequence is proposed to run in a tangential pattern, with markers for deep neocortical neurons in the hippocampus and arcopallium, while the neuronal markers of superficial or middle neocortical layers are present in the derivatives of the hyper-, meso- and nidopallium. Furthermore, Suzuki et al. (2012) observed that unlike *in vivo*, where progenitors appear to have a more restricted fate, cultured chick neural progenitors of either medial or lateral pallium produce multiple layer-specific neuronal subtypes in the same chronological sequence as seen in mammals. Thus, a temporal sequence of differentiation of neuronal types comparable with that found in the mammalian neocortex is apparently inherent in parts of the avian pallium (Suzuki et al., 2012; see also Chen et al., 2013; Nomura et al., 2013a,b). Nomura et al. (2008) did make a controlled regional analysis of their markers in both species, finding a topographic parallelism in the lower layer markers but not in the upper layer markers (see **Table 1**). However, the report by Suzuki and Hirata (2013) can be criticized for not having sampled developmentally equivalent regions in birds and mammals, as these authors seem to imply that mammalian neocortex can be compared straightforwardly with the whole pallium of sauropsids. Furthermore, they did not document with precision the sites of the explanted tissues, which may cast some doubts on the *in vivo*–*in vitro* comparisons.

Another report (Dugas-Ford et al., 2012) used a set of cross-species reliable markers of neocortical input and output layers in different mammalian species (see Rowell et al., 2010), and observed that the superficial layer IV (thalamo-recipient layer) markers EAG and RORB were found in the anterior dorsal cortex (area D2) of the turtle and in the thalamo recipient hyperpallium, entopallium and Field L of birds. On the other hand, the above mentioned Er81, marker for the efferent neocortical layer V, was expressed in the posterior two thirds of the dorsal cortex of the turtle, with some scattered neurons anteriorly (note the discrepancy with Nomura et al., 2008; Suzuki et al., 2012; **Table 1**). There was a small region of overlap, but no double-stained neurons were found. In birds, Er81 was found in the hyperpallium and arcopallium, which is partly consistent with the findings by Nomura et al. (2008). Dugas-Ford et al. (2012) claim that a basic pallial circuit comprising input-thalamorecipient and output neurons is present in the ventral as well as in the dorsal pallial regions of both mammals and sauropsids. These authors further argue that their findings support Karten’s homology hypothesis; however, this evidence does not contradict the claustroamygdalar hypothesis either (Aboitiz, 1995; Striedter, 1997; Puelles et al., 2000), as all neocortical input-output elements might just correspond to those found in the avian hyperpallium and turtle dorsal cortex. Furthermore, a tangentially separated input-output organization, similar to that found in the nido-arcopallium, is observed in the mammalian amygdala, where thalamorecipient neurons in the lateral anterior amygdala express RORB and EAG, while Er81 is expressed in output neurons of the basolateral amygdala (see Allen Brain Atlas). Note that the basolateral amygdala and the lateral anterior amygdala derive from the lateral and ventral pallium, respectively; Medina et al. (2011). This evidence is in general consistent with homology between the nido-arcopallium and the mammalian amygdala, as the claustroamygdalar hypothesis prescribes.

Dugas-Ford et al.’s (2012) article has sparkled some debate in the controversy about neocortical homologues. Reiner (2013) celebrated these findings, as they bridge evidence from expression markers and hodology. This author comments the evidence that markers of layers IV and V are also expressed in the mammalian amygdala. Nonetheless, he restates the argument for homology between the thalamic projections to the visual extrastriate/auditory cortex in mammals, and those to the field L2 (auditory) and entopallium (visual) in birds, based on similarities in the auditory input to the auditory cortex and the nidopallium (both tonotopic), while the auditory projection to the mammalian amygdala is non-tonotopic. In addition, he calls attention to some similarities in gene expression between the avian nidopallium and the auditory cortex, which are positive for dbx1 and negative for Lmo4, while the claustroamygdaloid complex of mammals shows the reverse pattern.

On the other hand, Medina et al. (2013) assert that some of the markers used by Dugas-Ford et al. (2012) are not specific to the layers they are referred to, and are expressed in other parts of the pallium and even the subpallium (see above). Furthermore, these are likely general markers of thalamorecipient and descending projection pallial neurons. However, these authors leave open the possibility of a different kind of homology, termed genetic or deep homology, between the phenotypes observed in the different groups, which implies a genetic conservatism underlying the development of non-homologous structures (Shubin et al., 2009).

Two very recent articles used bioinformatic profiling of some 50 constitutive genes (Jarvis et al., 2013) and 16 developmentally regulated genes (Chen et al., 2013) in the zebrafinch brain. Among the pallial developmentally regulated genes are Pax6, Emx2 and others that are important for early patterning, as well as later-expressed ones like Er81 and COUP-TF2. Nonetheless, these authors did not use other markers that distinguish between the ventral pallium and other pallial regions, like Emx1 or Lhx9 (Puelles et al., 1999, 2000; Medina and Abellán, 2009; Medina et al., 2011). Jarvis et al. (2013) and Chen et al. (2013) observed a distinct partitioning of gene expression profiles, in which the mesopallium and the hyperpallium dorsale (HD) appear as a single subdivision, while the more dorsal hyperpallium apicale/hyperpallium apicale lateralis (HA, lHA) on one hand, and the nidopallium on the other, express very similar patterns of gene expression. Finally, the intercalated hyperpallium (IH) and the thalamorecipient entopallium (visual) and field L (auditory) also share a distinct molecular profile (see color codes in **Table 1**). Based on this pattern, these authors have proposed a new nomenclature for the subdivisions of the avian brain, in which there is a semi-mirror reversal of gene expressions in both directions away from the intermediate mesopallial lamina. These authors speculate about a close developmental relation between the dorsal and ventral pallium, which is proposed to be a continuous field in early development but a topographic distortion and/or a dorsal migration would separate this field into the dorsal hyperpallia apicale and apicale lateralis in a dorsal position, and the nidopallium in a ventral position. They make also some considerations about a possible functional columnar-like organization of the avian pallium.

The findings by Jarvis et al. (2013) and Chen et al. (2013) were critically commented by Montiel and Molnár (2013), who in first instance showed concerns about the selection of the genetic markers used, and whether using a different set of genes would have provided different results. Montiel and Molnár (2013) assert that some constitutive genes (such as the 21 glutamate receptor genes) are very likely overrepresented in the sample and may not refer to developmental patterning processes. Furthermore, Chen et al. (2013) did not use any early markers that distinguish between different pallial regions in several species (Brox et al., 2004; Medina et al., 2005, 2011), which makes it difficult to assess homologies of the distinct pallial components in other vertebrates. Thus, Montiel and Molnár (2013) highlight the differences with other gene mapping and cell migration studies (Fernández et al., 1998; Redies et al., 2001; Nomura et al., 2008; Medina and Abellán, 2009; Medina et al., 2011; see also Puelles et al., 1999, 2000), and propose that similarity in gene expression profiles between say, the nidopallium and parts of the hyperpallium, could more easily be explained by the parallel or convergent recruitment of similar developmental programs in these regions (see also Aboitiz, 2011; Medina et al., 2013). In this context, the relatively late developmental appearance of the apical hyperpallial markers in Chen et al. (2013; see their Figure 20) might relate to the fact that the dorsal pallium is a newly expanding region in birds, while the genetically similar nidopallium and mesopallium already started its amplification in the reptilian lineage as the DVR. It will be of the greatest interest to observe similar studies performed in reptiles, mammals and also amphibians to determine which of these patterns are ancestral and which derived in amniotes.

Finally, Belgard et al. (2013) published a much larger scale transcriptomic analysis (over 5,000 genes) in the telencephalon of adult mouse and chicken, obtaining different results. In chicken, samples were taken from hyperpallium, mesopallium, dorsolateral corticoid area, nidopallium and arcopallium. Mouse samples were dissected from layers of dorsal and lateral cortex, claustrum-endopiriform complex and pallial amygdala. Striatum and hippocampus from both species were also used as control samples. First, regions of known homology and conserved function across species like the hippocampus and corpus striatum showed a significant conservation of expression domains. Secondly, these authors found a somewhat weaker but still significant correlation between expression patterns in neocortical layer IV of the mouse and in the nidopallium of the chicken (**Table 1**). Other components did not show significant correlations. However, this finding was not interpreted as resulting from homology between neocortical layer IV and nidopallium, but from convergence due to functional and hodological similarity, because (i) only 5 genes explained the cross-species overlap; (ii) three of the “top hub” genes display expression in a variety of brain regions; and (iii) these regions have a different embryonic origin (nidopallium, ventral pallium; neocortical layer IV, dorsal pallium), and their findings did not evidence any trace of tangential migration from the mammalian ventral pallium into the neocortex.

Despite some dicrepancies across studies which might partly be due to the use of different bird species, an overall pattern shows up when examining these findings altogether (see **Table 1**). The avian medial pallium and the medial aspect of the hyperpallium on one hand, and the arcopallium on the other, appear to express markers of early produced and mid-term hippocampal, neocortical and amygdalar neurons in mammals. On the other hand, regions in the avian hyperpallium, nidopallium and mesopallium display mid-term or late-produced mammalian neocortical markers. The general pattern observed is that in both mammals and birds, there is little topographic overlap between the mammalian inferior layer and the superior layer markers in most pallial regions, with the obvious exception of the mammalian neocortex, and the developmentally equivalent avian hyperpallium (**Table 1**; but in birds these have not yet been found to be clonally related as in the neocortex). The mesopallium and nidopallium notoriously lack inferior layer markers, showing much similarity with the mid-and superior layer neocortical phenotypes.

To end this section, we want to emphasize that if the question is the origin and evolutionary divergence of the amniote brain, research might be better directed to the reptilian rather than to the avian brain, particularly to the reptilian DVR and the dorsal cortex, derivatives of the ventral and dorsal-medial pallium, respectively (Luzzati et al., 2009; Nomura et al., 2013a; Northcutt, 2013). The reptilian condition is closer to that of the ancestral amniote than that of birds, and the possibility of convergence due to an increased brain size in birds and mammals may be less significant with reptiles. In addition, the study of amphibian brain development might provide important insights about the ancestral amniote brain (Brox et al., 2004).

## A UNIFYING HYPOTHESIS

**FIGURE 3:**
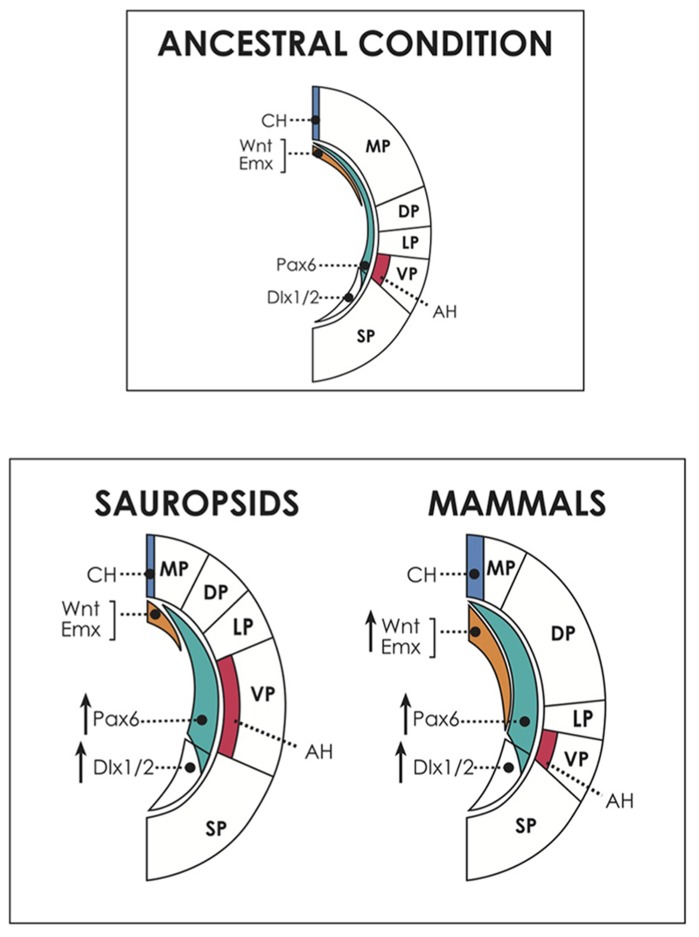
**Above, dorsal and ventral patterning centers in the cerebral hemispheres, and presumed ancestral condition.** The dorsally located cortical hem (CH) expresses dorsalizing factors like Wnts and Emxs and patterns the embryonic medial pallium (MP, hippocampal formation and homologous structures) and the dorsal pallium (DP, neocortex in mammals; dorsal cortex/hyperpallium in sauropsids). On the other hand, the antihem, induced by Pax6 activity, specifies the ventral pallium (pallial amygdala in mammals, DVR/nidopallium in sauropsids). Pax 6 is expressed in a anteroventral-to-caudodorsal gradient that counteracts with the dorsalizing factors, and contributes also to neocortical and hippocampal patterning in mammals. In the common ancestor, perhaps similar of present-day amphibians, there was possibly a relatively large dorsal pallium, at the expense of the development of other pallial regions (Northcutt, 2013). Below, hypothetical scenario of developmental evolution in the pallium of amniotes. Pax6 is proposed here as a candidate to drive the amplification of progenitor proliferation in the brains of different amniotes, but there may be other or additional factors contributing to this process. Pax6 expression is proposed to have been upregulated in both sauropsids and mammals. In reptiles, this event produced a modest amplification of the antihem (AH) and the ventral pallium (VP), giving rise to the dorsal ventricular ridge. In birds, Pax6 amplification reached higher levels, expanding the nidopallium and mesopallium, and also reaching the DP, contributing to generate the hyperpallium. Conversely, in mammals, in addition to Pax6 enhancement there was a concomitant upregulation of dorsal signals (illustrated by an increase in Wnt and Emx activities), which antagonized Pax6 signaling, restricting the expansion of the antihem. Furthermore, in mammals, upregulation of Pax6 and dorsal signals show a significant overlap, allowing Pax6 to influence the expansion of the DP, giving rise to the neocortex. Not shown for simplicity is the anterior forebrain, patterned by the action of FGFs, which may have also contributed to brain expansion particularly in mammals. Note that the subpallium also increased in size in all amniotes. SP, subpallium, marked by the expression of markers like Dlx1/2.

Attempting to interpret some of the apparently discrepant evidences shown above, we proposed a neuro-developmental model of the amniote brain, taking into account the recent evidence of the patterning effect of distinct and evolutionarily conserved morphogenetic centers, located in the dorsomedial hemisphere (the cortical hem), the anterior and ventral forebrain (anterior neural ridge-related to olfactory placodes, and septal region in later stages), and the lateral hemisphere (the antihem; **Figure 3**; Hoch et al., 2009; Medina and Abellán, 2009; Aboitiz, 2011; Alfano and Studer, 2013). As most of the evidence on these signaling centers and their activity has been collected in the mouse neocortex, we will make a brief summary of these studies in order to provide the appropriate context. When referring to other brain regions or to nonmammalian specie it will be clearly stated.

The cortical hem in the dorsomedial hemisphere expresses signaling molecules like Gli3, BMPs, Wnts and the downstream genes Emx1/2, in a posteromedial-to-anterolateral decreasing gradient which specifies the medial and the dorsal pallium (Hoch et al., 2009; Medina and Abellán, 2009). As mentioned, the cortical hem is also the source of most reelin-producing Cajal-Retzius neurons. Interestingly, both Wnts and reelin have been found to contribute to the radial arrangement of the neocortex, and to the differentiation of pyramidal-like cell types (Zhou et al., 2004; Nomura et al., 2008; Abellán et al., 2010). Opposing this signaling wave is Pax6, expressed at high levels in the anterolateral hemisphere (including the subpallial lateral ganglionic eminence) and gradually decreasing expression in the dorsomedial direction (Stoykova et al., 2000). This gene contributes to establish a boundary between the dorsal hemisphere and the corpus striatum (Georgala et al., 2011a). Furthermore, Pax6 specifies the antihem, which is coextensive with the ventral pallium and secretes EGFs, FGFs and frizzled-related proteins (FRPs) that counteract the influence of dorsal signals like Wnts (Assimacopoulos et al., 2003). Pax6 and the antihem participate in the differentiation of pallial amygdalar structures (Cocas et al., 2011), and Pax6 is required for proper development of both ventral and dorsal pallial structures in mammals (for more detailed reviews involving these and additional regulatory systems see Hoch et al., 2009; Medina and Abellán, 2009; Medina et al., 2011, and Alfano and Studer, 2013).

The third signaling center that has been identified is the anterior neural ridge, which develops into the commisural plate after closure of the neural tube, which secretes FGFs in a rostral-to-caudal decreasing gradient and is responsible for the differentiation of the ventral telencephalon, frontal cortex and the anterior telencephalic midline. It is also critical in regulating dorsalizing signals deriving from the cortical hem (Monuki and Walsh, 2001; Hoch et al., 2009; Suzuki-Hirano and Shimogori, 2009; Alfano and Studer, 2013). FGFs are required for the development of olfactory structures and sexual differentiation, characters that are developmentally related (Wilkie, 2005). Furthermore, amplification of anterior forebrain centers expressing FGFs may have been involved in the origin of the cerebral hemispheres and the differentiation of olfactory structures in vertebrates (Aboitiz and Montiel, 2007a,b).

In neocortical development, Emx2, Pax6 and FGFs act in counterbalance. Pax6 deficiency results in an areal expansion of visual cortex, a reduction of frontal areas, and dysgenesis of limbic structures (Stoykova et al., 2000; Tole et al., 2005). On the other hand, Emx2 deficiency produces an expansion of frontal regions and a reduction of occipital regions (visual cortex; Bishop et al., 2000). Finally, Wnts/Emx2 and FGFs play opposing roles in the differentiation of the frontal cortex, the former inhibiting and the latter promoting its development (Fukuchi-Shimogori and Grove, 2001; Shimogori et al., 2004). Therefore, there are at least three partly opposing morphogenetic gradients that combine to pattern the dorsal hemisphere into a medial pallium (hippocampal formation), with strong expression of Gli3, Wnts and other factors; a lateral and ventral pallium (amygdala and olfactory cortex) under the predominant influence of Pax6 and FRPs, and a dorsal pallium that receives a strong influence from the hem, the antihem and the anterior forebrain. These interacting patterning centers are highly conserved in evolution, being present in agnathans (Retaux and Kano, 2010).

While dorsal morphogens like Wnt have a profound mitogenic effect in early neocortical development, their influence gradually vanishes and becomes replaced by Pax6 activity (Zhou et al., 2006; Machon et al., 2007; Kuwahara et al., 2010), which promotes neural stem cell self-renewal and the consequent production of intermediate progenitors. Nonetheless, the latter undergo a developmental program supressing Pax6 activity and expressing Tbr2, Svet 1 and other factors (Englund et al., 2005; Sansom et al., 2009). Although it is suppressed in intermediate progenitors, Pax6 is a critical promotor of intermediate progenitor production, as its mutation or downregulation produces deficits in the intermediate progenitors, while its overexpression results in a substantial increase and self-renewal of these elements (Warren et al., 1999; Tarabykin et al., 2001; Nieto et al., 2004; Quinn et al., 2007; Pontious et al., 2008; Wen et al., 2008; Sansom et al., 2009; Jia et al., 2011). Thus, Pax6 is required for the development of some relatively early, layer V phenotypes (like those expressing Er81; Tuoc and Stoykova, 2008), but in later development it facilitates the production of intermediate progenitors in the SVZ, and promotes the differentiation of late born superficial neocortical layers. Additionally, Pax 6 is required for the development of limbic structures, as its mutation results in profound deficits in the development of the amygdaloid complex (Tuoc et al., 2009; Cocas et al., 2011; Georgala et al., 2011a,b).

Considering this evidence, we proposed that an upregulation of progenitor proliferative activity and the consequent production of intermediate progenitors, might have resulted in the generation of a SVZ and the amplification of the progenitor pool in the ancestral mammalian neocortex, as well as in the reptilian DVR and more intensely in the avian mesopallium, nidopallium and hyperpallium (**Figure 3**; Aboitiz, 2011; Aboitiz and Montiel, 2012). At this point, a good candidate to play a role in this amplification process is the gene Pax6, which as said is expressed in the whole pallial neuroepithelium and has a dose-dependent effect on progenitor proliferation. Another candidate is Notch, which regulates the cell cycle to balance stem cell maintenance with neurogenesis (see below; Ables et al., 2011; Nomura et al., 2013b). In sauropsids, this expansion may have occurred in two steps: a moderate increase in reptiles gave rise to the DVR in the ventral pallium (although no SVZ is yet discernible in this structure; Northcutt, 2013), while in birds a more substantial upregulation of Pax6 or related factors resulted in the development of a SVZ supporting amplification of the nido/mesopallium in the ventral/lateral pallium, and of the hyperpallium in the dorsal pallium. Consistent with this proposal, a SVZ has been associated with the generation of superficial neocortical layers and with late generated components of the avian nido/mesopallium, as well as in the hyperpallium of some birds (see above; Charvet et al., 2009; Cheung et al., 2010; Aboitiz, 2011). Of note, since Pax6 promotes expression of Er81 in some layer V neocortical neurons (Tuoc and Stoykova, 2008), it is possible that the Er81-positive output neurons observed in the mammalian hippocampus and amygdala, as well as in the sauropsidian medial and ventral pallium (Nomura et al., 2008; Dugas-Ford et al., 2012; Suzuki et al., 2012) are also specified by relatively early Pax6 activity. Furthermore, the *in vitro* evidence of a common differentiation sequence in clonal progenitors in the neocortex and in different pallial regions of birds (Suzuki et al., 2012) fits the concept that despite originating in different embryonic regions, the avian nido-mesopallium and the mammalian neocortex share a common program for progenitor amplification and neural differentiation (Aboitiz, 2011). This program may derive from a conserved gene activation network for pallial development that was present in the common ancestor of both groups.

Nonetheless, the above does not account for the striking anatomical and embryological differences between the brains of mammals and sauropsids: the DVR/meso-nidopallium of birds originates largely in the ventral pallium, with a non-columnar architecture (although columnar-like circuits have been recently described in birds; Ahumada et al., 2009; Wang et al., 2010; Chen et al., 2013), in absence of pyramidal cell morphologies, while the neocortex originates in the dorsal pallium and displays a conspicuous organization with pyramidal neurons arranged in a clonally related columnar array. Thus, in the sauropsidian pallium, the antihem may have suffered a significant amplification as a consequence of Pax6 upregulation, but the dorsal cortical hem maintained a reduced size. Consequently, the ventral pallial DVR and avian nidopallium would largely be derivatives of an amplified antihem (presumably due to upregulation of Pax6 or other factors), but at the same time there would be a limited differentiation and proliferation of dorsal pallial elements (see also Puelles, 2001). In fact, the cortical hem is more rudimentary in sauropsids than in mammals (Medina and Abellán, 2009; Abellán et al., 2010) and cortical hem-derived Wnt factors, as well as reelin, are much more weakly expressed in the chicken than in the mouse (Garda et al., 2002). Likewise, the cortex of the Madagascar gecko has recently evidenced an extremely lower rate proliferation and neural differentiation than other amniotes (Nomura et al., 2013b). In addition, these authors observed that Notch signaling, which constraints the self-renewal potential of progenitor cells by facilitating neural differentiation (Lakomá et al., 2011), is upregulated in the developing gecko cortex compared to mammals and birds. However, different neuron subtypes are generated sequentially during development, as in birds and mammals. These results provide support to the concept that similar changes in the regulation of cortical progenitors have been involved in the expansion of different brain regions in amniotes, and also opens the possibility that Pax6 may have worked in concert with other signaling cascades to increase or decrease progenitor proliferation.

In mammals on the other hand, there was an amplification of the dorsal signals emanating from the cortical hem. The cortical hem-derived elements reelin (secreted by Cajal-Retzius cells) and Wnts and other factors contribute to the maintenance and differentiation of a radial glia scaffolding and the establishment of a columnar organization (see above). Thus, in the mammalian pallium, the increasing influence of dorsal-derived factors may have limited the expansion of the antihem (ventral pallium), which according to Fernández et al. (1998) becomes obliterated in late developmental stages. Nonetheless, Pax6 activity was also upregulated in the pallium. In the neocortex, Pax6 activity participates in the generation of output neurons (Er81 positive, hippocampal and neocortical layer V neurons) but also facilitates the generation of a SVZ and the differentiation of late-produced elements in the superficial layers. In this context, the population of ventral pallial neurons described above, that transiently populate the developing neocortex and stimulate the proliferation of late cortical progenitors, may be under the direct or indirect influence of Pax6 (Teissier et al., 2010, 2012) which as said is required for ventral pallial development (Assimacopoulos et al., 2003).

Summarizing the points discussed, in early and mid- developmental stages, low or moderate levels of proliferative activity (possibly driven by low levels of Pax6 or other factors promoting progenitor division) result in the development of markers corresponding to early born and mid-term neurons in the neocortex (layers V and IV, respectively), in the amygdala and in the hippocampus (predominantly early born in the latter) of mammals. Likewise, in reptiles and birds these markers are present in the hippocampus, dorsal cortex/Wulst, and in the meso-nidopallium and arcopallium (see **Table 1** and Medina et al., 2011). With increasing proliferative activity (by upregulation of Pax6 or other factors), we suggest that there is a fundamental amplification in the mammalian neocortex and in the avian meso-nidopallium and hyperpallium, resulting in the amplification of the SVZ and the further differentiation of mid-term and late born neurons in these structures (especially neocortical layers IV, II and II, and meso-nidopallium in birds; **Table 1**). One remaining question concerns the apparent absence of early phenotypes in the mesopallium and nidopallium of birds. One possibility is that these phenotypes are related to the time of onset of neurogenesis, which might have started in later stages in this region, or that this developmental pathway is truncated by some unknown mechanism (Suzuki and Hirata, 2013). As mentioned above, the expansion of different embryonic components in mammals and sauropsids (dorsal pallium vs. ventral pallium, respectively), would result from differences in the influence of dorsal patterning factors, which in mammals became upregulated and restricted the expansion of the antihem, while in sauropsids there was a much weaker inhibition from dorsal centers to the expanding antihem.

Finally, a less investigated player in this context is the anterior neural ridge, which secretes FGFs like the antihem and counteracts the action of dorsal morphogens, contributing to the differentiation of the frontal cortex (Shimogori et al., 2004; Aboitiz and Montiel, 2007a,b; Alfano and Studer, 2013). An upregulation of FGFs may have been important for brain expansion in all amniotes, but in early mammals this was possibly crucial for the amplification of the olfactory system and the differentiation of the frontal cortex (Aboitiz and Montiel, 2007a,b; Aboitiz, 2011). Noticeably, a mammalian-specific diencephalic FGF8 enhancer has been recently identified close to the FGF8 genes, which faithfully replicates the pattern of FGF8 expression during development (Nakanishi et al., 2012). In fact, FGF8 expression is much lower in chick than in mouse diencephalon. Diencephalic patterning may influence neocortical development via thalamo-cortical axons (Suzuki-Hirano and Shimogori, 2009; Molnár et al., 2012), but it is also possible that additional enhancers are related to cortical and olfactory expansion in the mammalian lineage.

## PUTTING IT ALL TOGETHER: SENSORY AND ECOLOGICAL ADAPTATIONS DRIVING BRAIN EXPANSION

As mentioned in the Introduction, the origin of mammals is marked by profound and complex restructuring of the reproductive habits, metabolism, and sensory development. This is not unique for mammals: in reptiles and especially in birds, there was also a serious reorganization of metabolism, locomotory and reproductive habits, concomitant with a notorious increase in brain size (Northcutt, 2013). Nonetheless, birds remained closer to the reptilian design in several aspects, including brain organization. What factors drove the divergence in patterning processes between sauropsids and mammals that were outlined above? This is a critical evolutionary question, for which we still do not have a clear answer. Nonetheless, a scenario will be proposed in which behavioral and sensory adaptations may have channeled natural selection for the origin of the mammalian and avian brains.

Mammals were characterized by profound changes in sensory capacities, many of them associated with nocturnal life (vision, audition) and homeothermy (somatic sensation, olfaction). Mammals have night-adapted eyes and have developed a strong degree of binocularity (Heesy and Hall, 2010). Interestingly, binocularity and nocturnal vision have been associated to a development of the lemnothalamic visual pathway –directed to the dorsal pallium- in both mammals and sauropsids (Heesy and Hall, 2010; Gaillard et al., 2013). Consequently, both mammals and binocular birds have a well developed dorsal pallium (neocortex and hyperpallium, respectively). In addition, there was an emphasis in somatic sensation associated to the loss of scales, the appearance of hair follicles and skin mechanoreceptors, muscle spindles and joint receptors that make up the somatosensory system and is accompanied by the development of a powerful cortico-spinal tract that participates in fine motor control (Aboitiz and Montiel, 2007a,b; Northcutt, 2011; Rowe et al., 2011). Both, the development of lemnothalamic vision and the amplification of the somatic sensory input were very likely facilitated by mutations favoring expansion of the dorsal pallium.

Concerning the so-called collothalamic pathways, the enhancement of auditory capacity by the development of a curved cochlea allowed detection of high-frequency sounds, and the middle ear ossicles that increased tympanic impedance of airborne vibrations (Luo et al., 2011). On the other hand, the tectofugal visual pathway is likely to have been reduced by virtue of its relevance to color vision and diurnal behavior, which were not the choice of early mammals. In these conditions, one likely possibility is that collothalamic afferents were redirected into the dorsal pallium, perhaps driven by the transient migratory pathway from ventral to dorsal pallium referred to above, which also contributed to the expansion of the dorsal pallial neuroepithelium (Teissier et al., 2010).

Furthermore, and possibly more important, olfaction is one of the most expanding senses in mammals, which is verified by the extensive olfactory receptor gene family in this group, being at least 10-fold larger than that of any other vertebrate group (Niimura, 2009), and the olfactory epithelium increases some other 10 times in size with the development of turbinal bones (Rowe et al., 2011). Olfaction is critical for social behavior in most mammals (and other vertebrates), via both the main and accessory olfactory systems. Individual recognition, scent marking, sexual behavior and mother-child bonding are all strongly dependent on olfaction. In mammals, the accessory and main olfactory systems are importantly connected with areas involved in social reward, modulating neuroendocrine functions that facilitate social learning and maternal behavior (Sánchez-Andrade and Kendrick, 2009; Schaal, 2010). This condition may have been critical for a change in the lifestyle of early mammals, enabling them to establish close social bonds and to increase maternal investment beyond what is found in reptiles. For example, mother-pup bonding and lactation are strongly olfactory-dependant in most mammals (Lévy et al., 2004; Schaal, 2010). Furthermore, olfactory circuits are critical for orienting behavior and spatial memory in all vertebrates, especially mammals (Jacobs, 2012). Perhaps taking advantage of a socially driven olfactory expansion, such circuits were probably exploited by early mammals to make predominantly olfactory-based maps of space, labeling routes and places and detecting predators and conspecifics. These spatial maps depend largely on the hippocampus, which in mammals contains a multimodal representation to generate episodic and spatial memories (Ergorul and Eichenbaum, 2004; Eichenbaum and Robitsek, 2009). The dorsal cortex of reptiles bears functional resemblance to the entorhinal and subicular cortices that in mammals interfaces between the hippocampus and the neocortex. Furthermore, although the reptilian dorsal cortex receives a visual projection, it does not participate directly in vision but rather supports learning and memory (Powers, 1990, 2003). Thus, the development of olfactory-dominated space maps may have benefited significantly from an expansion of the dorsomedial pallium, which began to incorporate other sensory modalities (like auditory and visual colothalamic) as it expanded (Aboitiz et al., 2003). In fact, despite its columnar development and sensory input-output organization, the mammalian neocortex displays important similarities with the olfactory cortex both in its intrinsic sircuitry (Shepherd, 2011) and a widespread tangential, associative organization that is shared with the ancestral reptilian cortex (Lynch, 1986). Furthermore, the olfactory cortex projects both directly and indirectly into the orbitofrontal cortex, which participates in conscious smell perception and regulates motivated and social behavior (Lynch, 1986; Shepherd, 2011). Thus, a discrete expansion of polymodal orbitofrontal regions for processing chemical and other stimuli might have also facilitated and regulated exploratory behavior.

In this context of ecological and behavioral adaptations, the emergence of a laminar neocortex by the amplification of proliferative signals originating in both the dorsal and the lateral hemisphere, facilitated the development of new behavioral and sensory abilities that were crucial in establishing the mammalian lifestyle. This is not to say that the mammalian neocortex is in any way superior in sensory or cognitive processing than say, the avian brain; in fact, there is ample evidence of highly complex cognitive capacities in many birds. It is thus more likely that birds acquired these sophisticated capacities independently of mammals.

On the other hand, the line of early sauropsids was characterized by the acquisition of scales (feathers in birds) that did not allow fine touch, limiting somatosensory processing. Likewise, most reptiles did not develop a powerful respiratory system and had a limited sense of olfaction. Likewise, audition was not particularly developed as noted by the lack of auditory specializations in reptiles and in many birds compared to mammals. In these circumstances, the most powerful sensory system was vision, which as in lower vertebrates, is directed mainly to mesencephalic centers. Thus, the default trend was to develop the collothalamic pathway, which became directed to the ventral pallium, together with the other important sensory modality audition. Only in some birds that have developed frontal vision and have nocturnal habits, has the lemnothalamic visual pathway and the related hyperpallium aquired a significant development (Heesy and Hall, 2010; Gaillard et al., 2013). The moderate expansion of the reptilian brain (mainly the DVR) compared to amphibians, was associated to important changes in exploratory and social behavior (Northcutt, 2013). In the line leading to birds (maniraptoran dinosaurs), there was a further trend to increase brain growth that was not exclusive of aves, which has been associated to the development of motor coordination abilities required for flight (Balanoff et al., 2013). In addition, other factors like maternal behavior, spatial orientation and memory capacity, that became increasingly required as the individual home range expanded dramatically due to flight behavior, may have been significant selective factors as well. Thus, there may be some ecological parallels in the evolutionary brain development of mammals and birds (maniraptors), namely the development of maternal behavior and the reliance on precise spatial maps, although the sensory systems involved in each group were different (olfaction in mammals, vision in birds).

## FINAL COMMENT

The evolution of the amniote brain consists of the diversification of distinct morphologies by the differential modulation of highly conserved morphogenetic fields. In this sense, there is a shared patterning mechanism underlying the telencephalic development of all amniotes (and possibly other vertebrates), in which the production of neuronal phenotypes and circuits is determined by specific genetic programs that were acquired very early in evolution, and have been put to use in different different contexts in each lineage to make up similar networks within diverging morphologies (see Aboitiz and Montiel, 2007b). The factors driving the modulatory shifts in different taxa derive from selective pressures posed by particular ecological conditions, which in mammals were marked by nocturnality, new reproductive habits, and the associated changes in social behavior. In this sense, there is a deep conservatism underlying the apparent structural diversity of vertebrate brains.

## Conflict of Interest Statement

The authors declare that the research was conducted in the absence of any commercial or financial relationships that could be construed as a potential conflict of interest.
